# The effect of dyslipidemic drugs on mortality: A meta-analysis

**DOI:** 10.4103/0253-7613.48878

**Published:** 2009-02

**Authors:** Shuchi Jain, Vikas Vaishnavi, Bhaswat S. Chakraborty

**Affiliations:** Research and Development, Cadila Pharmaceuticals, Ahmadabad, Gujarat, India

**Keywords:** All-cause mortality, cardio-vascular disease mortality, coronary artery disease mortality, dyslipidemic, meta-analysis

## Abstract

Most of the previously published meta-analyses include studies exploring the effect of statins, rather than all dyslipidemic drugs, on mortality. We explored the overall effect of all dyslipidemic drugs on coronary artery disease mortality, cardio-vascular disease mortality and all-cause mortality. A meta-analysis of all randomized controlled trials that were published before February 2006 was carried out. Data sources were published articles in bibliographic electronic databases and medical journals. The article selection criteria included all randomized placebo-controlled trials of at least one year duration and those which measured at least one of the following clinical endpoints: coronary artery disease mortality, cardio-vascular mortality or all-cause mortality. Information on sample size, follow up period, drug used, and clinical outcomes was abstracted independently by two authors. Disagreements were resolved by consensus. The meta-analysis (19 trials, 59033 patients) showed a significant relative risk reduction of coronary artery disease mortality of 23% (*P*<0.00001), cardiovascular disease mortality of 19% (*P*<0.00001) and all-cause mortality of 14% (*P*<0.0001), without any significant heterogeneity and inconsistency between the trials. It was concluded from this meta-analyses that dyslipidemic drugs are indeed highly effective medicines and confer benefit to patients, in terms of primary and secondary prevention of coronary artery disease.

## Introduction

Cardio-vascular disease (CVD) is the world's leading killer, accounting for 16.7 million or 29.2% of the total global deaths in 2003. While deaths from heart attacks have declined more than 50% since the 1960s in many industrialized countries, 80% of the global CVD related deaths now occur in low and middle-income nations, which cover most countries in Asia. In India, in the past five decades, the rate of coronary disease among urban populations has risen from 4 to 11%.[1]

Dyslipidemia (elevated total cholesterol and Low-density Lipoprotein (LDL), and depressed High-density Lipoprotein (HDL) levels are independent risk factors. Statins (HMG-CoA reductase), fibrates and resins are known dyslipidemic drugs, which act by increasing or decreasing lipoprotein (Lp) or its components levels. Many trials have been conducted to show their effectiveness in treating Coronary Artery Diseases (CAD). But very few have shown statistically significant results.

Thus, many meta-analyses have been done to verify the findings of trials involving various dyslipidemic drugs. However, most of these studies include trials of statins only; if the studies included other dyslipidemic drugs also, dietary and other kinds of interventions were also compared, along with the dyslipidemic drugs. Meta-analyses including all the dyslipidemic drug trials only are very few.

The effect of these drugs on all-cause mortality is still unclear.[2–4] Thus, this meta-analysis was done to evaluate the effectiveness of dyslipidemic drugs on CAD mortality, CVD mortality or all-cause mortality.

## Materials and Methods

### Eligibility Criteria

Two authors separately reviewed the abstracts produced by the literature search to identify studies that are randomized placebo-controlled trials evaluating the effectiveness of any dyslipidemic drug and reporting one of the following clinical endpoints: CAD mortality, CVD mortality or all-cause mortality. The follow up period should have been greater than or equal to one year.

The most frequent reasons for study exclusion were study duration being less than one year, trial being nonrandomized or non placebo-controlled, two or more drugs being compared to a single placebo in the same study or clinical endpoints not being reported. Studies in which eligibility criteria included a particular lifestyle disease, e.g.: hypertension[5] or diabetes were excluded. Studies published in languages other than English and abstract form only were also excluded.

### Study Identification

Strategies to identify studies included an electronic search of bibliographic databases (Clinical Trials, Cochrane Reviews, PubMed and Science Direct), electronic as well as manual search of medical journals (American Journal of Cardiology, Annals of Internal Medicine, Archives of Internal Medicine, British Medical Journal, Circulation, European Heart Journal, Lancet, The Journal of the American Medical Association and The New England Journal of Medicine), consultation with the experts, review of reference lists from eligible trials and use of the “See Related Articles” feature for key publications in PubMed (March 2006).

This search resulted in the identification of 20 eligible studies, which are listed along with their characteristics in Table 1. However, due to increase in heterogeneity (funnel plot), one study (ACAPS) was excluded from the final results, even though it fulfilled the inclusion/exclusion criteria. Thus, the results summarize meta-analysis of 19 studies.

**Table 1:** Characteristics of the trials selected in this article

*Study characteristics*	*Drug used*	*Year of publication*	*Mean follow up duration (years)*	*Number of subjects (Intervention/Control)*	*Mean age (years)*	*Percentage of male subjects*
WOSCOP	Pravastatin	1995	4.9*	3302/3293	55	100
VA-HIT	Gemfibrozil	1999	5.1	1264/1267	64	100
LIPID	Pravastatin	1998	6.1	4512/4501	62	83
CARE	Pravastatin	1996	5	2081/2078	59	86
AFCAPS/TexCAPS	Lovastatin	1998	5.2 *	3304/3301	58	57.5
MARS	Lovastatin	1993	2.2	134/136	58*	91
PROSPER	Pravastatin	2002	3.2 *	2891/2913	75.3	48.3
NEWCASTLE	Clofibrate	1971	3.7	244/253	52.3	80.5
FLORIDA	Fluvastatin	2002	1	265/275	60.5	83
PLAC-I	Pravastatin	1995	3	206/202	57	37.9
PLAC-II	Pravastatin	1995	3	75/76	63	-
4S	Simvastatin	1994	5.4	2221/2223	59	81.5
MAAS	Simvastatin	1994	4	193/198	30-67	-
CCAIT	Lovastatin	1995	2	165/166	54.9	81
ACAPS#	Lovastatin	1994	2.8	460/459	61.7	51.5
KAPS	Pravastatin	1995	3	224/223	57	100
REGRESS	Provastatin	1995	2	450/435	56.2	100
HPS	Simvastatin	2002	5	10269/10267	-	75.25
LIPS	Fluvastatin	2002	3.9†	844/833	60	83.8
CIS	Cholestyramine	1984	5*	59/57	46.1	81

WOSCOP = West of Scotland Coronary Prevention Study;[6] VA-HIT=Veterans Affairs High-Density Lipoprotein Intervention Trial Study;[7] LIPID=Long-Term Intervention with Pravastatin in Ischemic Disease;[8] CARE=Cholesterol and Recurrent Events;[9] AFCAPS/TexCAPS=Air Force/Texas Coronary Prevention Study;[10] MARS=Monitored Atherosclerosis Regression Study;[11] PROSPER=Prospective Study of Pravastatin in the Elderly at Risk;[12] NEWCASTLE=Trial of Clofibrate in IHD;[13] FLORIDA=Fluvastatin on Ischemia;[14] PLAC-I=Pravastatin in Reduction of Atherosclerosis;[15] PLAC-II=Pravastatin Limitation of Atherosclerosis in the Coronary Arteries;[16] 4S=Scandinavian Simvastatin Survival Study;[17] MAAS=Simvastatin Multicentre Anti-Atheroma Study;[18] CCAIT=Canadian Coronary Atherosclerosis Intervention Trial;[19] ACAPS=Asymptomatic Carotid Artery Progression Study;[20] KAPS=Kuopio Atherosclerosis Prevention Study;[21] REGRESS=Regression Growth Evaluation Statin Study;[22] HPS=Heart Protection Study;[23] LIPS=Lescol Intervention Prevention Study;[24] CIS=Coronary Intervention Study.[25]

* Average

† Median

# This study was dropped from the meta-analysis due to an increase in heterogeneity, even though it fulfilled the inclusion/exclusion criteria.

### Data Collection

Full articles have been reviewed for eligibility. For articles meeting the inclusion criteria, relevant data was extracted and entered in the evidence table.

### Statistical Analysis

Meta-analysis was performed using the Mantel-Haenszel method for fixed effect model and Der-Simonian and the Laird method for random effect model. Graphs of the outcomes for included trials were examined visually and by using the Chi-square test to identify heterogeneity in the outcome variables across different studies. Because the results of our meta-analysis did not vary depending on whether the fixed or random effect model was used, random effect results are presented. The results are displayed as summary odds ratio (OR), relative risk (RR) and 95% confidence interval (CI) of OR and RR for CAD mortality, CVD mortality and all cause mortality. I^2^ value is also calculated. An I^2^ value represents the percentage of the total variation across trials, which is due to heterogeneity rather than chance, and is considered thus: I^2^ value <25% is low and >75% is high. All analyses is done using RevMan 4.2.8 (Cochrane Collaboration).

## Results

**Figure 1:**
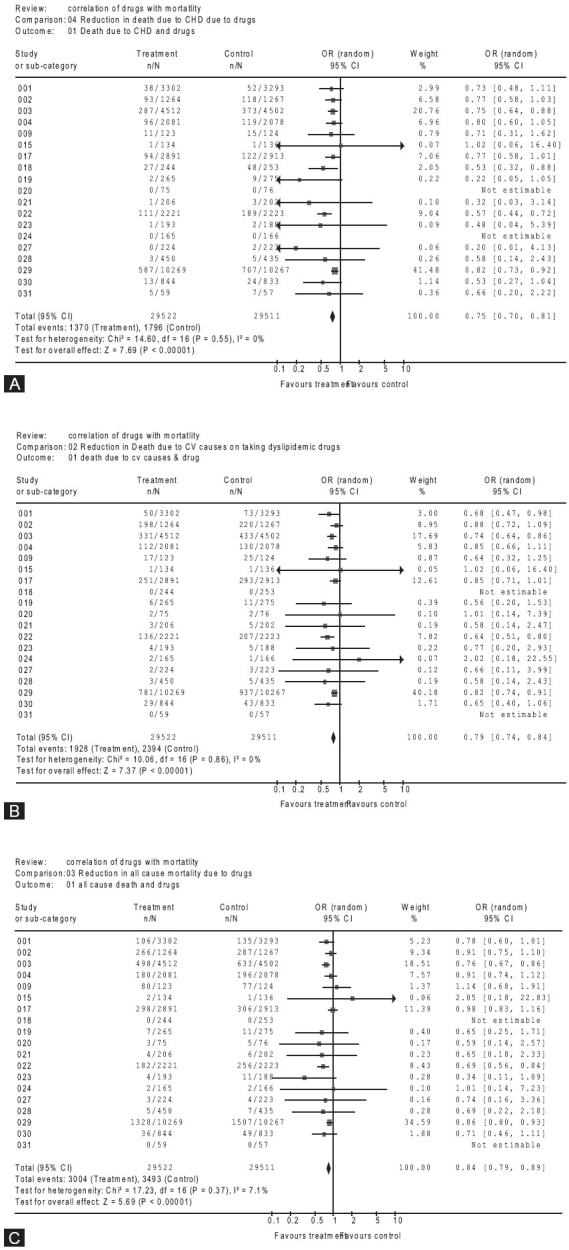
Effect of dyslipidemic drugs (compared with placebo) on odds of a) CAD mortality; b) CVD mortality and c) all-cause mortality. For References, compare the patient numbers in both arms in Table 1. The treatment and control columns present the number of deaths by the total number of randomized patients in that arm. The weight represents the relative weight given to the study. The final column gives the point estimate of odds ratio and its 95% confidence interval.

**Figure 2:**
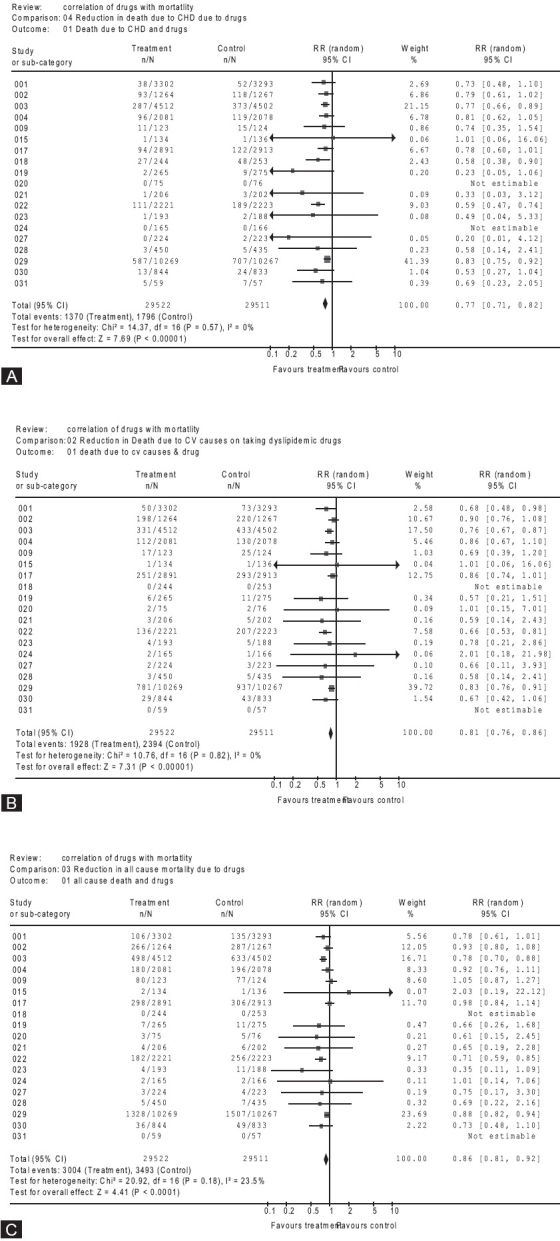
Risk of a) CHD mortality, b) CVD mortality and c) all-cause mortality, in the treatment group, compared to that in placebo. For References, compare the patient numbers in both arms in Table 1. The treatment and control columns present the number of deaths by the total number of randomized patients in that arm. The weight represents the relative weight given to the study. The final column gives the point estimate of relative risk and its 95% confidence interval.

Figures 1 and 2 show the effect of treatment with dyslipidemic drugs on CAD mortality, CVD mortality and all-cause mortality. The point estimate and 95% CI of OR and RR are reported. For all studies, combined patients who received treatment had a statistically significant advantage for all-cause mortality, along with CAD mortality and CVD mortality. Treatment reduced the relative risk of CHD mortality by 23% (summary relative risk is 0.77, 0.71 to 0.82), compared with placebo. For CVD mortality and all-cause mortality, reduction was by 19% (summary relative risk is 0.81, 0.76 to 0.86) and 14% (summary relative risk is 0.86, 0.81 to 0.92) respectively. The corresponding summary OR for CHD was 0.75 (95% CI: 0.70 to 0.81); for CVD the summary OR was 0.79 (95% CI: 0.74 to 0.84) and that for all- cause mortality was 0.84 (95% CI: 0.79 to 0.89) showing a reduction of 25, 21 and 16%, respectively. In each of these analysis, the results of Chi-square test for heterogeneity are not significant (*P*>0.10) and the value of I^2^ (I^2^<25%) suggests a fairly low amount of inconsistency across the trials included.

## Discussion

This meta-analysis shows that lipid lowering drugs reduce the relative odds of CHD mortality, CVD mortality and all-cause mortality by about 15-25%. The population in these trials includes men, women, elderly people, hypercholesterolemic and patients with normal cholesterol levels.

The conclusions drawn in this meta-analysis are different from that of Pignone *et al.*,[4] who found that the effect of statins on all-cause mortality is insignificant. The differences are possibly because 1) Pignone *et al*, limited their analysis to the statin trials only. 2) They included only primary prevention trials. Unlike their study, in this meta-analysis, studies of fibrates and resins have been also included. Apart from this, primary, secondary as well as mixed type trials have been included in this meta-analysis. But these conclusions are similar to that drawn by Ross *et al.*,[26] who found that statins are effective in reducing CVD mortality, along with all-cause mortality (OR = 0.76, 0.67 to 0.86).

The differences are due to the exclusion of studies in which a second drug was added if the targeted levels of cholesterol were not achieved, in our meta-analysis.

Generalization of these findings to other populations – such as Asians – is difficult, as trials selected for the analysis do not adequately represent this population. Generally, the population that participated was of European or American descent.

The strength of this meta-analysis is that it is based on more than 50,000 randomized patients. However, the short comings of this analysis are: 1) it was not based on individual data but available data from the literature; 2) some of the articles could not be included as they were unavailable; 3) absolute risk was not calculated.

Future research can be done to know the effectiveness of dyslipidemic drugs on people of non European origin. Further research can be done to calculate absolute values to strengthen the findings in this analysis.
